# The Emerging Role of Biotechnological Drugs in the Treatment of Gout

**DOI:** 10.1155/2014/264859

**Published:** 2014-04-16

**Authors:** L. Cavagna, W. J. Taylor

**Affiliations:** ^1^Division of Rheumatology, University and IRCCS Policlinico S.Matteo Foundation, Viale Golgi 2, 27100 Pavia, Italy; ^2^Rehabilitation Teaching and Research Unit, Department of Medicine, University of Otago Wellington 6242, New Zealand

## Abstract

One of the most important therapeutic advances obtained in the field of rheumatology is the availability of the so-called bio(techno)logical drugs, which have deeply changed treatment perspectives in diseases such as rheumatoid arthritis and ankylosing spondylitis. According to the steadily increasing attention on gout, due to well-established prognostic and epidemiology implications, in the last 5 years, the same change of perspective has been observed also for this disease. In fact, several bio(techno)logical agents have been investigated both for the management of the articular gout symptoms, targeting mainly interleukin-1**β**, as well as urate-lowering therapies such as recombinant uricases. Among the IL-1**β** inhibitors, the majority of studies involve drugs such as anakinra, canakinumab, and rilonacept, but other compounds are under development. Moreover, other potential targets have been suggested, as, for example, the TNF alpha and IL-6, even if data obtained are less robust than those of IL-1**β** inhibitors. Regarding urate-lowering therapies, the recombinant uricases pegloticase and rasburicase clearly showed their effectiveness in gout patients. Also in this case, new compounds are under development. The aim of this review is to focus on the various aspects of different bio(techno)logical drugs in gouty patients.

## 1. Introduction

Gout is an autoinflammatory disease associated with increased blood levels of urate and due to deposition of monosodium urate crystals in and around joints [1]. Over recent decades, the prevalence of this condition is steadily increasing and gout is becoming one of the most common causes of inflammatory arthritis in industrialised countries [2–7]. In fact, joints are the typical target of the disease and articular gout attacks are between the most painful conditions described [8]. But gout and hyperuricemia may also affect the kidneys [9] and cardiovascular system [10] and are frequently complicated by the metabolic syndrome [11]. Gout burden is substantial: joint flares, tophi, polyarticular involvement, and chronicization deeply impact patients' quality of life and workability [12–16], whereas gout by itself is an independent risk factor for cardiac and all-cause mortality [17, 18]. Current treatment is first based on lifestyle measures and then on a pharmacological approach [19, 20]. Recently, several biotechnological drugs have been employed and approved for gout treatment. This review is focused on the analysis of these treatments that potentially could reduce gout burden and the unmet needs of its pharmacological approach.

## 2. Gout Pharmacological Treatment: Targets of Bio(techno)logical Drugs

Gout pharmacological treatment is aimed at relieving articular symptoms and reducing hyperuricemia [19, 20]. Both targets are of primary importance and should be achieved in gouty patients. In the last years, several bio(techno)logical drugs have been found effective for these purposes.

Symptomatic relievers and urate-lowering therapies (ULTs) act on different pathways. Symptomatic relievers mainly target IL-1, a proinflammatory cytokine that has been linked to gout since late 1980s [21] and is now widely accepted as central to the initiation of the inflammatory cascade that culminates in gouty arthritis. In particular, the activation of NALP3 inflammasome by uric acid crystals increases the production of IL-1 and the inflammatory state [22]. The understanding of these mechanisms thus opened a new perspective in acute and chronic gout management [23].

Even if IL-1 is pivotal in gout, we should consider that also other inflammatory cytokines could be potentially involved; in particular, previous studies showed that also TNF*α* [24–29] and interleukin-6 (IL-6) [28–30] are overexpressed in patients with gouty arthritis. The role of TNF*α* in gout is also suggested by the increased expression of soluble TNF receptors I and II (sTNFR-I/II) in synovial fluids from gouty patients during arthritis resolution phases [31]. The blockade of TNF*α* and IL-6 through biotechnolological drugs is well established and routinely performed in rheumatoid arthritis (RA) [32–34] and, limiting to TNF*α*, in ankylosing spondylitis (AS) [35], with a large literature in terms of treatment survival, side effects, and warnings [36, 37]. Recently, several authors underlined the central role of T lymphocyte in the appearance of gout articular damage [38–40] in particular through the upregulation of RANKL [39] and thus of osteoclastogenesis [41]. On this basis, also T cells targeting should be considered a potential treatment of gouty arthritis. Finally, we should also consider that increased levels of transforming growth factor (TGF*β*1) [42, 43] and interleukin-10 [42] are typically found in the synovial fluid of patients during gouty arthritis resolution.

Although ULTs target several steps of the human enzymatic breakdown of uric acid as well as the renal system to increase urate urinary excretion, from the bio(techno)logical point of view, one of the most intriguing targets is an enzyme that humans lost due to a gene missense mutation: uricase [44]. This enzyme converts urate to allantoin, a substance more water-soluble and thus more readily eliminated than urate. The possibility of reducing serum urate levels by means of the action of an enzyme lost by humans during primate evolution is very fascinating, almost like an inversion of the evolutive process.

## 3. Symptoms Relievers Bio(techno)logical Drugs: IL-1 Inhibitors

### 3.1. Canakinumab

Canakinumab is a fully human, anti-IL-1*β* monoclonal antibody first approved for the treatment of cryopyrin associated periodic syndrome [45].

The effectiveness of canakinumab in acute gout was first reported in 2010 in a phase-2 dose ranging trial of 8 weeks [46]. Enrolled patients were randomized to receive a single dose of subcutaneous canakinumab (10, 25, 50, 90, or 150 mg; *n* = 143) or intramuscular triamcinolone acetonide (40 mg; *n* = 57). After 72 hours, a dose-related pain reduction was observed in canakinumab group for every dosage used. Moreover, canakinumab 150 mg was more effective than triamcinolone acetonide in every timepoint considered (e.g., 24, 48, and 72 hours and 4, 5, and 7 days after treatment—*P* < 0.05 in all cases), also reducing the risk of subsequent articular flares (relative risk reduction 94% for canakinumab 150 mg versus triamcinolone acetonide). The overall incidence of adverse events, generally mild or moderate in severity, was similar in both groups (41% and 42%, resp.).

Another study showed that the improvement of health-related quality of life (SF-36) was faster with canakinumab 150 mg compared to intramuscular triamcinolone acetonide 40 mg [47]. In another double-blind, double-dummy, dose-ranging study, involving 432 gout patients initiating allopurinol, a single canakinumab dose of 50–300 mg or 4-weekly dosing over 4 months was superior to colchicine (0.5 mg/day) in articular flares prophylaxis [48]. In particular, there was a 64% to 72% reduction in the risk of experiencing ≥1 flare for canakinumab doses ≥50 mg versus colchicine at 16 weeks (hazard ratio (HR): 0.28–0.36, *P* ≤ 0.05). No differences were observed among groups in terms of adverse events, the treatments being generally well tolerated.

The *β*-RELIEVED and the *β*-RELIEVED-II were two 12-week randomised, multicentre, active-controlled, double-blind, parallel-group studies with double-blind 12-week extensions which aimed to assess canakinumab effectiveness in acute flares and reflares of gouty arthritis [49]. Only patients with a recent articular gout acute flare (VAS pain ≥50 mm), with at least three other flares in the previous 12 months, and with contraindications to NSAIDs and/or colchicine were considered for study inclusion. The comparators were canakinumab 150 mg by subcutaneous injection and intramuscular triamcinolone acetonide 40 mg. A total of 456 patients were enrolled in the core set study (227 canakinumab, 229 triamcinolone acetonide) and 365 entered the extension study (174 canakinumab, 161 triamcinolone acetonide). With respect to triamcinolone acetonide, canakinumab significantly reduced mean 72-h VAS pain score (difference of 10.7 mm, *P* < 0.0001) as well as physician-assessed tenderness and swelling (OR 2.16 and 2.74, both *P* < 0.01). The efficacy of canakinumab with respect to triamcinolone acetonide was evident also in terms of delayed time to first new flare on both core (62% reduced risk of a new flare over 12 weeks) and extension studies (56% reduced risk of a new flare over 24 weeks) and median C-reactive protein levels reduction at 72 h and 7 days (OR 4.4 and 2.1, both *P* < 0.0001). Adverse events were observed in 66.2% of canakinumab-treated patients and in 52.8% of patients receiving triamcinolone acetonide. Infections were reported in 20.4% (canakinumab) and 12.2% (triamcinolone acetonide) of patients, being serious infections observed in 1.8% and 0%, respectively.

Based on these data, subcutaneous canakinumab has been approved by the European Medicines Agency for the on-demand symptomatic treatment of frequent gouty arthritis attacks in adult patients in whom NSAIDs, colchicine, or corticosteroids are contraindicated, not tolerated, or not effective [50] and is potentially useful also in acute gouty prophylaxis during ULT initiation [48].

Recently, canakinumab has been found effective as adjunctive treatment in patients with type I diabetes [51], and possibly also in patients with type 2 diabetes [52]. A large secondary prevention trial with canakinumab in patients with prior acute myocardial infarction is ongoing [53]. By considering the burden of metabolic syndrome [11] and cardiovascular complications [10] in gout patients, the results of these studies could be a further reason for the use of IL-1 inhibitors in this setting.

### 3.2. Rilonacept

Rilonacept (IL-1 TRAP) is a soluble decoy receptor Fc fusion protein that engages and inhibits both IL1*α* and IL1*β*. It is known also as a IL-1 Trap, because it is generated using Target-Related Affinity Profiling (Trap) technology [54]. Rilonacept has been first described as effective in gout in a 14-week, multicentre, nonrandomised, monosequence crossover study involving 10 patients. The active treatment period lasted 6 weeks, followed by other 6 weeks of withdrawal period that completed the study. Rilonacept was administered with a loading dose of 320 mg (two 2 mL injections) administered subcutaneously, followed by rilonacept 160 mg once a week. Only one patient withdrew from the study because of severe injection site erythema and induration. The remaining 9 patients significantly improved in terms of* patients' self-reported median pain visual analogue scale scores* and high-sensitivity C-reactive protein reduction; also two nonvalidated instruments, the* symptom adjusted* and the* severity-adjusted joint scores*, significantly improved, whereas no effects were observed on the number of affected joints.

Subsequently, further phase II and III studies have been performed. Phase II study involved 83 patients starting allopurinol (42 in the placebo group and 41 in the active treatment group). Rilonacept was administered subcutaneously once per week (loading dose 320 mg followed by 160 mg weekly) and the follow-up was 12 weeks [55]. Rilonacept significantly reduced the mean number of gout flares per patient during the entire follow-up (6 flares versus 33; *P* < 0.0011) and in particular during the first 4 weeks of follow-up (*P* < 0.007). The proportion of patients with articular flares during the study period was lower in the rilonacept than in the placebo group (14.6% versus 45.2%; *P* < 0.0037), whereas the most common adverse events were the occurrence of injection site reactions in patients treated with rilonacept.

The PRESURGE 1 was a 16-week follow-up phase III study involving 241 gout patients from USA and Canada with at least 2 previous articular flares and persistent hyperuricemia (>7.5 mg/dL). Concomitant to allopurinol treatment (300 mg/day), the patients were randomized to 16 once-weekly subcutaneous injections of placebo, rilonacept 80 mg, or rilonacept 160 mg, with a double (loading) dose on day 1. During the follow-up, the mean number of gout flares per patient was significantly reduced by rilonacept treatment, with respect to placebo (placebo: 1.06, rilonacept 80 mg: 0.29, rilonacept 160 mg: 0.21, *P* < 0.001 versus placebo). In particular, only 18.8% and 16.3% of patients had >1 gout flares, respectively, with rilonacept 80 and 160 mg: the differences with respect to placebo (46.8% of patients had >1 gout flares) were statistically significant (*P* < 0.001 for both). The number needed to treat (NNT) for the reduction of at least 1 gout flare was 2 for both rilonacept 160 and 80 mg groups [56].

The PRESURGE-2 was another phase III study 248 gout patients with the same selection criteria and treatment groups of the PRESURGE-1 study but involving patients from Germany, India, Indonesia, Republic of South Africa, and Taiwan [57]. In this study, both rilonacept dosages significantly reduced the occurrence of gout flares after the initiation of standard ULT, with >70% of patients having no flares. Similar to PRESURGE-1 study, safety and tolerability profile was acceptable with injection site reactions being the most common adverse events described in both rilonacept groups [56, 57].

By considering studies with active comparators, the effectiveness of rilonacept (320 mg at baseline) + indomethacin (50 mg* three times per day* for three days) after 72 hours was not superior to that of indometacin alone in terms of pain reduction in a group of 225 gout patients presenting within 48 hours since flare onset [58].

### 3.3. Anakinra

Anakinra is a recombinant human IL-1 receptor antagonist that differs from native human IL-1Ra because of the addition of a single methionine residue at its amino terminus [59]. Anakinra binds to both IL-1*α* and *β* [60] and is the first IL-1 inhibitor marketed and approved for RA treatment [61]. The first report on the effectiveness of this drug in gout has been published in 2007 [62]. In this open-labeled study, 10 patients with a long previous history of either recurrent gouty attacks or tophaceous gout that failed or not tolerated standard therapies were treated with anakinra and administered daily at a dose of 100 mg subcutaneously for 3 consecutive days. In all cases, treatment was rapidly effective and well tolerated during a short term follow-up (maximum: 2 months). Another retrospective study involving 10 patients with refractory gout showed less interesting results: in particular, 3 patients had a partial response and one was refractory to this treatment, whereas relapses were really common during follow-up [63]. This fact is not surprising if we consider the short half-life of anakinra and the short duration of treatment. One possible solution is the temporal prolongation of the treatment. In fact, further case reports confirmed this possibility, also in case of intermittent administration [64–67].

Anakinra has been tested in parallel with canakinumab as a potential treatment for type 1 diabetes patients [51]. Even if clinical trials on canakinumab in cardiovascular diseases are ongoing, more data are available for anakinra. In particular, anakinra improved left ventricular remodelling [68] and numerically lowered the incidence of heart failure [69] in patients with acute myocardial infarction and ST-segment elevation. IL-1 blockade with anakinra for 14 days significantly improved the aerobic exercise capacity of patients with heart failure with preserved ejection fraction and elevated plasma CRP levels [70]. On this basis, similar to canakinumab and rilonacept, also for anakinra, it is possible to suppose a wide range of positive effects in patients with gout. On this basis, similar to canakinumab and rilonacept, also for anakinra, it is possible to suppose a wide range of positive effects in patients with gout, by considering the impact of comorbidities (metabolic syndrome and cardiovascular disease) in the setting [10, 11].

When considering anakinra as a potential treatment for gout, some warnings must be regarded in particular in patients with severe renal failure [71]. In fact, anakinra clearance is diminished by 75% in patients with severe renal failure [72]. In these cases, the injections could be given at wider intervals [71]. Other common side effects are injection site reactions, upper respiratory tract infections, headache, nausea, and diarrhoea [73]. Also when considering these warnings, a recent study clearly showed not only the effectiveness but also the safety of anakinra in a cohort of 26 complex hospitalized patients with gout arthritis, 15 of them characterized by the occurrence of chronic renal disease [74].

Despite these findings and suggestions, to date, no clinical trials on anakinra in gout have been performed or proposed.

## 4. Symptoms Relievers Bio(techno)logical Drugs: Other Bio(techno)logical Drugs (Anti-TNF Alpha, Tocilizumab, Abatacept)

Literature data on these drugs are scanty. The first anti-TNF alpha used in gout arthritis was etanercept in 2004 [75]. The patient treated was a 53-year-old man with recurrent tophaceous polyarticular gout complicated by kidney involvement. After the failure of every treatment tried (colchicine, diclofenac, methylprednisolone, and opioids), etanercept (25 mg subcutaneously twice weekly) was started with subsequent reduction of gout attacks, painful joints, ESR, and CRP, also during ULT. Subsequently, Fiehn and Zeier [76] described another patient with refractory chronic polyarticular tophaceous gout successfully treated with infliximab (5 mg/kg I.V. at weeks 0, 2, 6 and then every 8th week). But treatment failure has also been reported, as recently described with infliximab [77].

IL-6 inhibitor tocilizumab (8 mg/kg/month) completely stopped gouty attacks in a 44-year-old man with a 12-year history of severe uncontrolled tophaceous gout refractory to colchicine and diclofenac [78]. Another drug currently used in RA [79], abatacept, completely suppressed gout activity in a patient with a long-term history of gout and subsequent superimposed RA [80]. Finally, although in 1996 Lioté et al. observed that the recombinant human and the ultrapure TGF*β*1 reduced the number of attacks in an experimental model of gout [81], no further studies on this potential therapeutic approach have been performed.

## 5. Urate-Lowering Bio(techno)logical Drugs

Even if nonrecombinant uricase from* Aspergillus flavus* was developed and used for reducing hyperuricemia in human tumour lysis syndrome in the late 1960s [82, 83], its use was complicated by production difficulties, the short half-life of the product, and the high frequency of severe allergic reactions (5%) [83]. Moreover, anaphylactic reactions may appear also in the long-term use of uricase, as evidenced in both animals [84] and humans [85] and repeated uricase injections can cause the production of antibodies that neutralise uricase enzyme activity [84, 85].

By considering its effectiveness in reducing serum urate levels, subsequent studies have been addressed to the identification of more stable and tolerated compounds.

### 5.1. Pegloticase

Pegloticase is a recombinant polyethylene glycol-conjugated form of uricase. With respect to the pure form of uricase, PEGylation should improve the half-life and reduce the immunogenicity of the enzyme [86]. Pegloticase was first tested via single subcutaneous injection in 13 gouty patients with high uricemia levels (>11 mg/dL) [87]. In the short term (7 days) dosages ranging from 4 to 24 mg led to normalization of urate levels in 11 cases. Patients treated with 8–24 mg of pegloticase had urate levels <6 mg/dL also after 21 days postinjection. In five subjects, the half-life and efficacy of pegloticase were reduced by the induction of antibodies, which, unexpectedly, were specific against the PEG residue rather than against the uricase itself.

Pegloticase was then tested intravenously on 24 patients with symptomatic gout (tophi, chronic synovitis, or flare within the past 6 months) and hyperuricemia (>7 mg/dL, not on conventional ULTs) [88]. The results of intravenous pegloticase were superior to those of subcutaneous injection for both hyperuricemia reduction and safety. The doses of 4 mg, 8 mg, and 12 mg reduced plasma urate concentrations to <2 mg/dL within 24 hours postinfusion, being the maximum decline (in average 10.2 mg/dL) obtained at 24–72 hours. With these doses, the results were satisfactory also 21 days after the infusion. Similar to other nonbio(techno)logical ULTs [89], gout flares and arthralgias were the main adverse events potentially linked to intravenous pegloticase [86], whereas subcutaneous delivery was frequently associated not only with injection site but also with widespread urticaria [85]. Interestingly, intravenous delivery seemed to be less immunogenic than the intramuscular one [86].

In a 16–18-week phase II study on 41 patients with gout, the highest proportion of patients who achieved and maintained the primary end-point (plasma urate level <6 mg/dL for at least 80% of the study period), the least pronounced increases in mean plasma urate levels between doses, and the highest proportion of time without hyperuricemia were obtained with the dose of 8 mg every 2 weeks, although all pegloticase doses were effective [90].

Two replicated, randomized, double-blind, placebo-controlled phase III trials (C0405 and C0406) were then conducted in patients with severe gout, refractory or intolerant to allopurinol, and serum uric acid concentration ≥8.0 mg/dL [91]. Two active treatment groups (Pegloticase 8 mg every 2 or 4 weeks) and one placebo group were planned. Prophylaxis against infusion-related reactions was given to all patients before each infusion (oral fexofenadine, 60 mg the evening before and again before infusion; acetaminophen, 1000 mg; and I.V. hydrocortisone, 200 mg, immediately before infusion). The primary end-point was the achievement of urate levels <6 mg/dL at months 3 and 6. A total of 225 patients participated: 109 in trial C0405 and 116 in C0406. When the 2 trials were pooled, the primary end-point was achieved in 36/85 patients in the biweekly group (42%; 95% CI, 32%–54%), 29/84 patients in the monthly group (35%; 95% CI, 24%–46%), and 0/43 patients in the placebo group (0%; 95% CI, 0%–8%; *P* < 0.001 for each comparison). Gout flares were reported in approximately 80% of patients across the 3 pooled study groups. Infusion-related reactions were the second most common adverse events, occurring, respectively, in 26%, 42%, and 5% of patients receiving pegloticase biweekly, monthly, and placebo, some cases fulfilling criteria for anaphylaxis. Serious infusion-related reactions occurred in 5% (pegloticase biweekly) and 8% (pegloticase monthly) of patients. Infusion-related reactions were the most common reason for pegloticase discontinuation during the study (10% for biweekly; 13% for monthly). The majority of patients (89%) developed high titers of pegloticase antibody, in particular, in patients experiencing infusion-related reactions (79%). Interestingly, the preliminary loss of urate-lowering efficacy preceded the first infusion reaction in 91% of biweekly pegloticase and in 71% of monthly pegloticase: low titres (≤1:2430) of anti-pegloticase antibodies were less likely to be associated with loss of ULTs response.

A more detailed analysis of tophi response in these two replicated trials with the subsequent open-label extension study was subsequently performed [92]. Tophi were observed in the majority of patients (73%), accounting for 547 visible localizations recorded at baseline. After 6 months of treatment, pegloticase 8 mg biweekly group had the complete resolution of at least one tophus without the appearance or the enlargement of any other tophus (e.g., complete response) in the 45% of cases (*P* = 0.002 versus placebo), in comparison to 26% of pegloticase 8 mg monthly and 8% of placebo group. In the three groups treatment, respectively, the 28%, the 19%, and the 2% of tophi had a complete response. The results were more relevant in patients with sustained urate-lowering response to therapy and steadily increased during the open-label extension. Furthermore, pegloticase markedly improved patients reported outcomes (Health-related Quality of Life and Physical Function), with the results being more strong in the biweekly treatment group with respect to monthly group [93]. The long-term safety (up to three years) was not substantially different from that described in the randomized phase [91].

Pegloticase is approved for the treatment of chronic gout in patients not responsive to conventional therapy in USA and for disabling tophaceous gout in patients who may also have erosive joint involvement in Europe. The treatment is scheduled intravenously at the dosage of 8 mg every 2 weeks over at least 2 hours. No dosage adjustments are required in older patients or in those with renal impairment. Premedication with antihistamines and corticosteroids is recommended, as well as the administration in a medical context. In G6PD deficient patients, all uricases are contraindicated due to the risk of haemolysis and methemoglobinaemia [94].

### 5.2. Rasburicase

Rasburicase is a recombinant uricase obtained from* A. flavus* approved in the early 2000s in USA and in Europe for the treatment of tumor lysis syndrome. Generally, this formulation is tolerated better than nonrecombinant urate oxidase. However, although potentially effective, up to now only few studies of rasburicase on gout have been performed.

Following the first case reports [95–97], Richette et al. [98] treated with intravenous rasburicase 10 patients with tophaceous gout, intolerant or refractory to allopurinol and suffering from moderate to severe chronic kidney disease. Five patients were treated daily for 5 days (group 1), as in tumour lysis syndrome [99], whereas the remaining five patients received 6 monthly injections of rasburicase (group 2). In all cases, premedication with 60 mg of methylprednisolone was administered. Even if group 1 had a rapid and marked decrease of serum urate levels, no differences were observed at 1 and 2 months with respect to baseline value; moreover, also tophi size did not change. Better results were observed in group 2, with the significant reduction of hyperuricemia after six infusions, and tophi size reduction in 2 cases. Adverse events were common, being observed in 8 out of 10 patients enrolled: 4 patients in group 1 and 2 in group 2 had a gouty articular flare, despite colchicine prophylaxis. Two patients in group 2 had an allergic reaction during the sixth infusion (bronchospasm and rash) requiring discontinuation of treatment.

Recently, some authors described the case of a patient with massive tophaceous gout that was concomitantly treated with 3 different ULTs, in particular allopurinol 600 mg/day, benzbromarone 100 mg/day, and 4 monthly rasburicase infusions that lead to the almost complete resolution of tophi before rasburicase withdrawal due to flushing and urticarial occurrence during the fifth infusion [100].

### 5.3. Bio(techno)logical Drugs under Development

Other symptoms relievers and ULTs bio(techno)logical drugs for gout treatment are under development. AC201 is an oral IL-1*β* inhibitor having also uric acid-lowering effects that is under evaluation in the prophylaxis against gout flares when initiating ULT [101]. Pegsiticase (Uricase-PEG 20, 3SBio, China) is another PEGylated derivative of a recombinant uricase from* C. utilis* [94]. Two phase 1 studies considering either the intramuscular [102] or the intravenous [103] administration of this compound for gout refractory to conventional therapy are ongoing.

## 6. Conclusions

In the last year, several bio(techno)logical drugs targeting particular points of gout and urate synthesis cascade have been approved for gout treatment by the US Food and Drug Administration and/or by the European Medicines Agency. As for RA and other rheumatology conditions, these drugs clearly opened a new era in the treatment of gouty patients, in particular in those with refractory disease or not tolerating conventional therapies. These drugs may act as symptom relievers or as ULTs. If IL-1 is the main target of symptomatic relievers agents (anakinra, canakinumab, and rilonacept), recombinant uricases (rasburicase and pegloticase) are the prototypical example of bio(techno)logical ULTs. Moreover, other bio(techno)logical compounds are at the pipeline.

By considering the burden of gout from the clinical and from the economic point of view [104], these new treatment possibilities may help the clinicians to improve patients' prognosis and impact. However, the high costs of these drugs clearly indicate that from the therapeutic point of view one of the most challenging points is patients' low adherence to gout therapies, with negative consequences on success rate and on disease progression [105]. In fact, there is also increasing interest by clinicians in the improvement of patient education, self-management training, and urate-lowering medication titration, in order to use the right drugs at the right moment and to provide the optimal gout care.
